# Persistent hypogammaglobulinemia after rituximab therapy in pediatric patients, prevalence and clinical outcomes

**DOI:** 10.1016/j.clicom.2025.04.001

**Published:** 2025-05-14

**Authors:** Susanna P.C. Höppener, Saskia R. Veldkamp, Mark C.H. de Groot, Saskia Haitjema, Julia Drylewicz, Jaap Jan Boelens, Caroline A. Lindemans, Joris van Montfrans, Annet van Royen-Kerkhof, Marc H.A. Jansen

**Affiliations:** a Pediatric Rheumatology and Immunology, Wilhelmina Children’s Hospital, University Medical Center Utrecht, Utrecht, the Netherlands; b Center for Translational Immunology, University Medical Center Utrecht, Utrecht University, the Netherlands; c Central Diagnostic Laboratory, University Medical Center Utrecht, Utrecht, the Netherlands; d Transplantation and Cellular Therapies, Memorial Sloan Kettering Cancer Center, New York, NY, USA; e Blood and Bone Marrow Transplantation, Princess Máxima Center for Pediatric Oncology, Utrecht, the Netherlands

**Keywords:** Hypogammaglobulinemia, Rituximab, Pediatric, B cell reconstitution

## Abstract

Hypogammaglobulinemia is a known side effect of rituximab (RTX) in adults, but its prevalence and persistence in children remain underexplored. This retrospective cohort study at a tertiary care center examines the prevalence and clinical outcomes of hypogammaglobulinemia in pediatric patients after RTX therapy. Patients aged ≤ 18 years treated with RTX for various indications between 2000 and 2020 were included. Patients were classified as having hypogammaglobulinemia when (1) IgG levels were <−2*SD* below reference for age, or (2) when they received immunoglobulin replacement therapy (IGRT) for the indication hypogammaglobulinemia. Hypogammaglobulinemia after RTX treatment was observed in 74/134 patients (55.2 %). Persistent hypogammaglobulinemia (>6 months) was observed in 46/91 patients (50.5 %), of whom 9 patients remained hypogammaglobulinemic >5 years. Low baseline IgG and IgM levels were significantly associated with persistent hypogammaglobulinemia, while patients receiving RTX therapy for autoimmune diseases were less frequently affected. CD19^+^ *B* cells reconstituted in a median of 11 months (*IQR*=[7.3–18.0]), while CD19^+^CD27^+^IgG^+^ switched memory B cells took significantly longer, with a median of 1.8 years (*IQR*=[1.0–2.9]). Three patients developed class-switch recombination-deficiencies and never recovered. Recurrent infections, of which two fatal, were recorded in 18 patients and were significantly more prevalent in those with persistent hypogammaglobulinemia. In conclusion, over half of children had low IgG levels and/or required IGRT for hypogammaglobulinemia following RTX therapy. Persistent hypogammaglobulinemia was associated with low pre-RTX IgG and/or IgM levels. Children with hypogammaglobulinemia after RTX are often IGRT-dependent, experience recurrent (and sometimes fatal) infections, and may develop secondary immunoglobulin class-switch defects.

## 1. Introduction

Rituximab (RTX) is a chimeric monoclonal antibody directed against CD20-antigen expressing B cells. The vast majority of B cells, however not plasma cells, express CD20 on their cell surfaces [1–3]. As a result, RTX therapy leads to significant B cell depletion. RTX therapy has proven to be effective against a wide variety of hematological malignancies and auto-immune diseases, including Systemic Lupus Erythematosus (SLE)), nephrotic syndrome, and post-hematopoietic stem cell transplantation (HSCT) complications (e.g. Epstein-Barr virus (EBV)-related lymphoproliferative disease, auto-immune hemolytic anemia) [1,4–10].

A possible side effect of RTX-mediated B cell depletion is hypogammaglobulinemia [11,12]. Since hypogammaglobulinemia is associated with an increased risk for infections and a higher mortality, it is essential to identify patients at risk for developing hypogammaglobulinemia [12–14].

While post-RTX hypogammaglobulinemia and its predictors are well-explored in adults, research on this complication in pediatric patients is limited [11–24]. With the rising use of RTX in children, alongside their developing immune systems and limited immune memory function in early childhood, it is important to gain more knowledge on the prevalence of post-RTX hypogammaglobulinemia and its clinical consequences in this population [25]. Moreover, data on (memory) B cell reconstitution after RTX therapy from a large pediatric cohort is lacking altogether, while this could give important insights into the effects of RTX on B cell memory and its recovery [13,24,26].

Therefore, this study aims to determine the prevalence, duration, clinical characteristics and outcomes of hypogammaglobulinemia in children after RTX therapy, to enable early identification, expectation management and treatment optimization for this patient category.

## 2. Methods

### 2.1. Patients and study design

This retrospective cohort study was conducted at a tertiary care center. All patients (aged ≤18 years) who received RTX between 2000 and 2020, were eligible for inclusion. Routine care data were collected and supplied pseudonymously by the Utrecht Patient Oriented Database (UPOD) [27]. Patients without at least one IgG measurement after the last RTX dose were excluded. To exclude hypogammaglobulinemia secondary to nephrotic syndrome, urinary protein levels were assessed in all patients. Patients with severe proteinuria (protein/creatinine ratio >300 mg/mmol with creatinine >2 mmol/L) and post-RTX hypogammaglobulinemia were excluded. A waiver of informed consent was granted by the regional medical ethics committee (METC Utrecht, 20–343/C).

### 2.2. Clinical data collection

Baseline characteristics including age, sex, Body Surface Area (BSA), and indication for RTX therapy at the time of first RTX administration were extracted from the patients’ electronic health records. Serum Ig levels (IgA, IgG and IgM), total blood B cell (CD19^+^) counts, and IgG^+^ switched memory B cell (CD19^+^CD27^+^) counts were extracted and expressed in standard deviations (*SD*) from age-matched reference values [28,29]. Urine protein and creatinine levels from spot and 24 h collections were also extracted. IgG measurements <1 year before RTX therapy were included as baseline. Baseline IgG measurements within 5 months after immunoglobulin replacement therapy (IGRT) were deemed unreliable and were excluded from analysis.

Pre-RTX hypogammaglobulinemia was defined as a serum IgG level <−2*SD* below reference for age before start of RTX therapy (accounting for IGRT as described above). Post-RTX hypogammaglobulinemia was defined as a serum IgG level <−2*SD* below reference for age or receiving IGRT for the indication hypogammaglobulinemia. Persistent hypogammaglobulinemia was defined as post-RTX hypogammaglobulinemia persisting >6 months after last RTX dose. B cell depletion pre- and post-RTX was defined as a peripheral cell count <−2*SD* below reference for age. The moment of IGRT-free normal IgG was defined as the first serum IgG measurement >−2*SD* for age, whilst not receiving IGRT. Moment of (IgG^+^ memory) B cell reconstitution was defined as first peripheral cell count >−2*SD* post-RTX.

RTX therapy characteristics (number of cycles and doses, and cumulative dose) were collected. One RTX cycle was defined as one or more doses with no more than 45 days between two doses. Single doses were calculated in mg/m^2^ using the BSA, and the cumulative dose was the sum of all single doses.

Use of immunosuppressive medication prior to and/or during RTX therapy was extracted and classified using the Anatomical Therapeutic Chemical (ATC) Classification System [30]. Individual chart review was conducted to assess time to recovery from hypogammaglobulinemia, IGRT dependency, IGRT indication, occurrence of Graft-versus-Host enteropathy, proteinuria, recurrent infections, and cause of death (when applicable). A patient was categorized as having recurrent infections when recurrent infections were documented by a treating physician after last RTX dose.

### 2.3. Statistical analysis

Descriptive statistics, including age, sex, baseline immunoglobulins (Igs), B cell counts, immunosuppressive medication history, and RTX therapy characteristics, were performed for the entire cohort and per RTX indication. Differences between RTX indications were assessed using chi-squared or Fisher’s exact tests for categorical variables and one-way ANOVA or Kruskal-Wallis tests for continuous variables. Outcomes included post-RTX hypogammaglobulinemia, persistent hypogammaglobulinemia, B cell recovery, and recurrent infections. Hypogammaglobulinemia rates pre- and post-RTX were compared using the McNemar test. For persistent hypogammaglobulinemia, incidence and predictors were determined. Baseline characteristics were compared between patients with and without persistent hypogammaglobulinemia using Mann-Whitney U tests for continuous variables and chi-squared or Fisher’s exact tests for categorical variables. We compared persistent hypogammaglobulinemia rates between RTX indications using Fisher’s exact test with post-hoc Bonferroni correction for multiple comparisons.

Longitudinal analysis of patients with hypogammaglobulinemia was performed. Kaplan Meier curves were fit for time to IGRT-free normal IgG levels, time to normal B cell counts, and time to normal IgG^+^ memory B cell counts. In time-to-event analyses, a minimal follow-up of 90 days after last RTX dose was required. Log-rank tests were used to compare survival curves between subgroups. Cox proportional-hazards models were used to identify variables associated with prolonged hypogammaglobulinemia, B cell, and IgG^+^ memory B cell reconstitution time.

Statistical significance was set at *p* < 0.05. R version 4.0.3 was used for statistical analysis (2020 the R Foundation for Statistical Computing).

## 3. Results

### 3.1. Patient characteristics

**Fig. 1.**
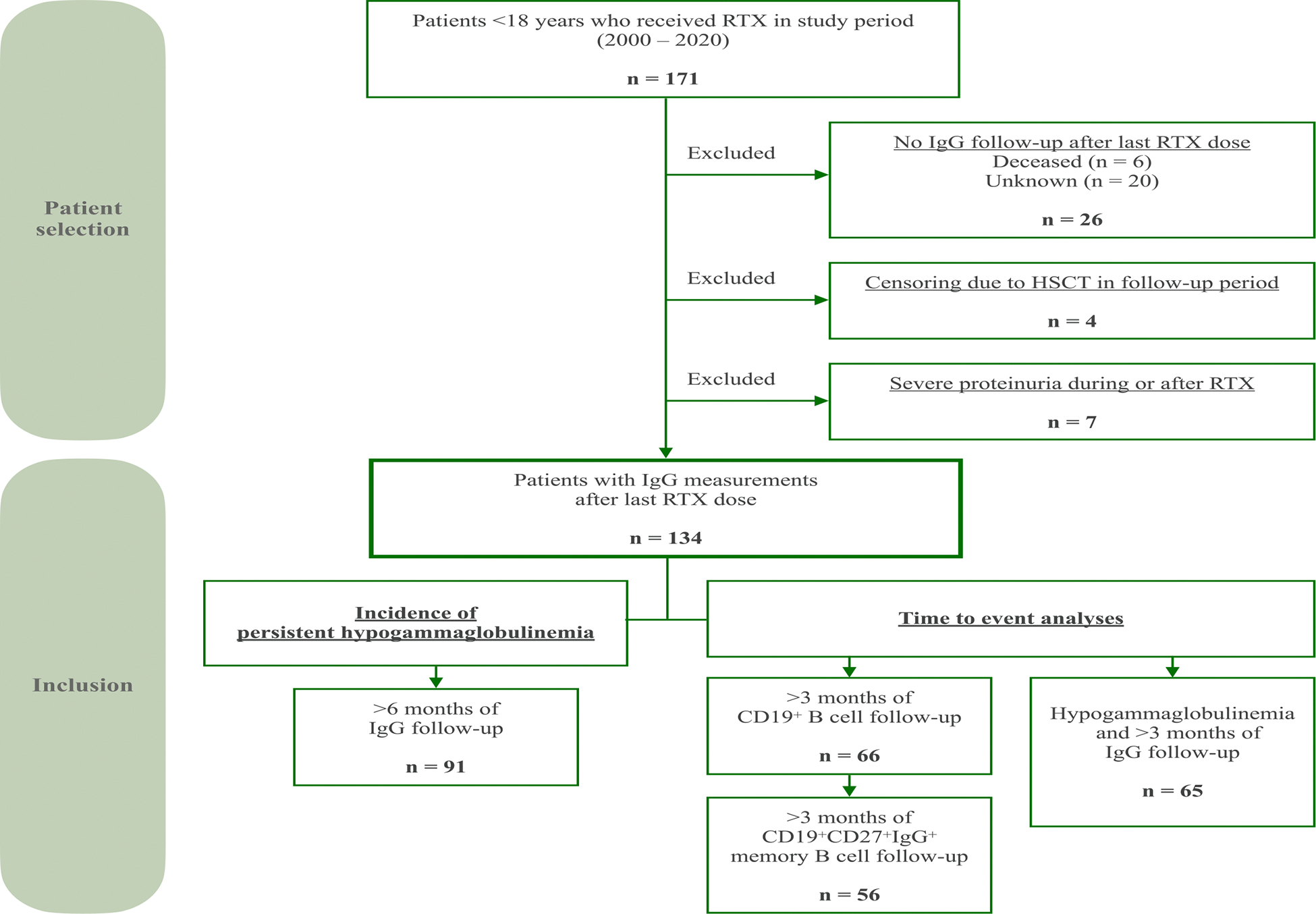
Flowchart depicting patient selection and inclusion. The upper part of the figure depicts patient selection. The lower part depicts inclusion in various analyses depending on availability of IgG and/or B cell measurements.

In total, 171 patients received RTX between 2000 and 2020 in one of the participating centers. Of these patients, 134 met the inclusion criteria (Fig. 1). Main reasons for exclusion were insufficient IgG follow-up data (*n* = 26), clinically significant proteinuria during or after RTX therapy (*n* = 7), and HSCT in the follow-up period (*n* = 4).

Baseline characteristics of included patients are presented in Table 1. Indications for RTX treatment were categorised in 3 groups: post-HSCT complication (*n* = 64), autoimmune diseases (*n* = 42) and miscellaneous (*n* = 28), with the latter grouping due to small sample sizes. Subgroup baseline characteristics per RTX indication are shown in Supplementary Table 1 and 2, respectively.

Regarding pre-RTX medication, a total of 50 patients (37.3 %) received IGRT within five months prior to RTX, with the highest occurrence in the post-HSCT group (54.7 %; *p* < 0.001) (Table 1, Supplementary Table 1). Other statistically significant differences between subgroups included age (*p* < 0.001), sex (*p* = 0.008), baseline IgG levels (*p* = 0.006), cumulative RTX dose (*p* = 0.023), and total CD19^+^CD27^+^ *B* cell counts (*p* = 0.011) (Table 1, Supplementary Table 1).

Prevalence of pre-RTX hypogammaglobulinemia was 25.2 % of all patients and differed significantly between RTX indications (*p* = 0.005; Table 2). The autoimmune diseases group had the lowest rate of pre-RTX hypogammaglobulinemia (15.6 %), whilst the highest occurrence was observed in the miscellaneous group (45.5 %), particularly among patients with nephrotic syndrome (75.0 %, Supplementary Table 1). Median IgG follow-up post-RTX was 1.5 years (*IQR* 0.4–4.2 years).

The 37 excluded patients were similar to the main cohort in age and indication for RTX (mean age 13.00 (SD=5.55), post-HSCT complications (*n* = 12), autoimmune diseases (*n* = 7), miscellaneous (*n* = 18).

### 3.2. Post-RTX hypogammaglobulinemia

The incidence of hypogammaglobulinemia rose significantly from 25.2 % (29/115) pre-RTX to 55.2 % (74/134) post-RTX (*p* < 0.001, Table 2). Additionally, 51.2 % (44/86) of patients with normal IgG levels before RTX developed new onset hypogammaglobulinemia.

Post-RTX hypogammaglobulinemia occurred most frequently in the miscellaneous group (75.0 %), mostly due to all patients with primary immunodeficiencies (PIDs) (*n* = 4) and nephrotic syndrome (*n* = 5) being affected (Supplementary Table 3). The lowest occurrence was observed in the autoimmune diseases group (23.8 %, Table 2).

As severe (grade III–IV) intestinal GvHD after HSCT may cause hypogammaglobulinemia through protein loss, it was assessed in all patients with hypogammaglobulinemia who received RTX in the HSCT setting. Severe intestinal GvHD was present in 8/74 cases.

IGRT was administered to 85/134 patients (63.4 %) during or after RTX therapy. Indications were diverse and often changed, but 37/85 (43.5 %) started IGRT solely due to low IgG levels. Other indications included prophylactic use (*n* = 16), treatment of allo- or autoimmune phenomena (*n* = 14), and combinations of the aforementioned (*n* = 18).

### 3.3. Persistent hypogammaglobulinemia

Persistent hypogammaglobulinemia (low IgG and/or IGRT for hypogammaglobulinemia >6 months post-RTX) occurred in 50.5 % (46/91) of patients with available measurements (Table 2). Among those with normal IgG pre-RTX, 41.0 % (25/61) developed hypogammaglobulinemia persisting >6 months post-RTX.

Persistent hypogammaglobulinemia rates differed significantly between groups (Table 2, *p* = 0.004), with the highest prevalence observed in the miscellaneous group. Post-hoc analysis revealed that children treated with RTX for autoimmune diseases had 0.13 times the odds of developing persistent hypogammaglobulinemia compared to those in the miscellaneous group (95 %-CI=[0.03 – 0.54], Bonferroni *p* = 0.004). In addition, baseline IgG levels were significantly lower in children who developed persistent hypogammaglobulinemia (median=−1.67*SD, IQR*=[−2.40;−0.90]) compared to those who did not (median=−0.85*SD, IQR*=[−1.69; −0.28], *p* < 0.001). This was also the case for baseline IgM levels (median=−1.47*SD, IQR*=[−1.98; −0.70] versus median =−0.40*SD, IQR*=[−1.26; 0.58], *p* = 0.004), but not for baseline IgA levels (*p* = 0.095).

### 3.4. Hypogammaglobulinemia recovery

**Fig. 2.**
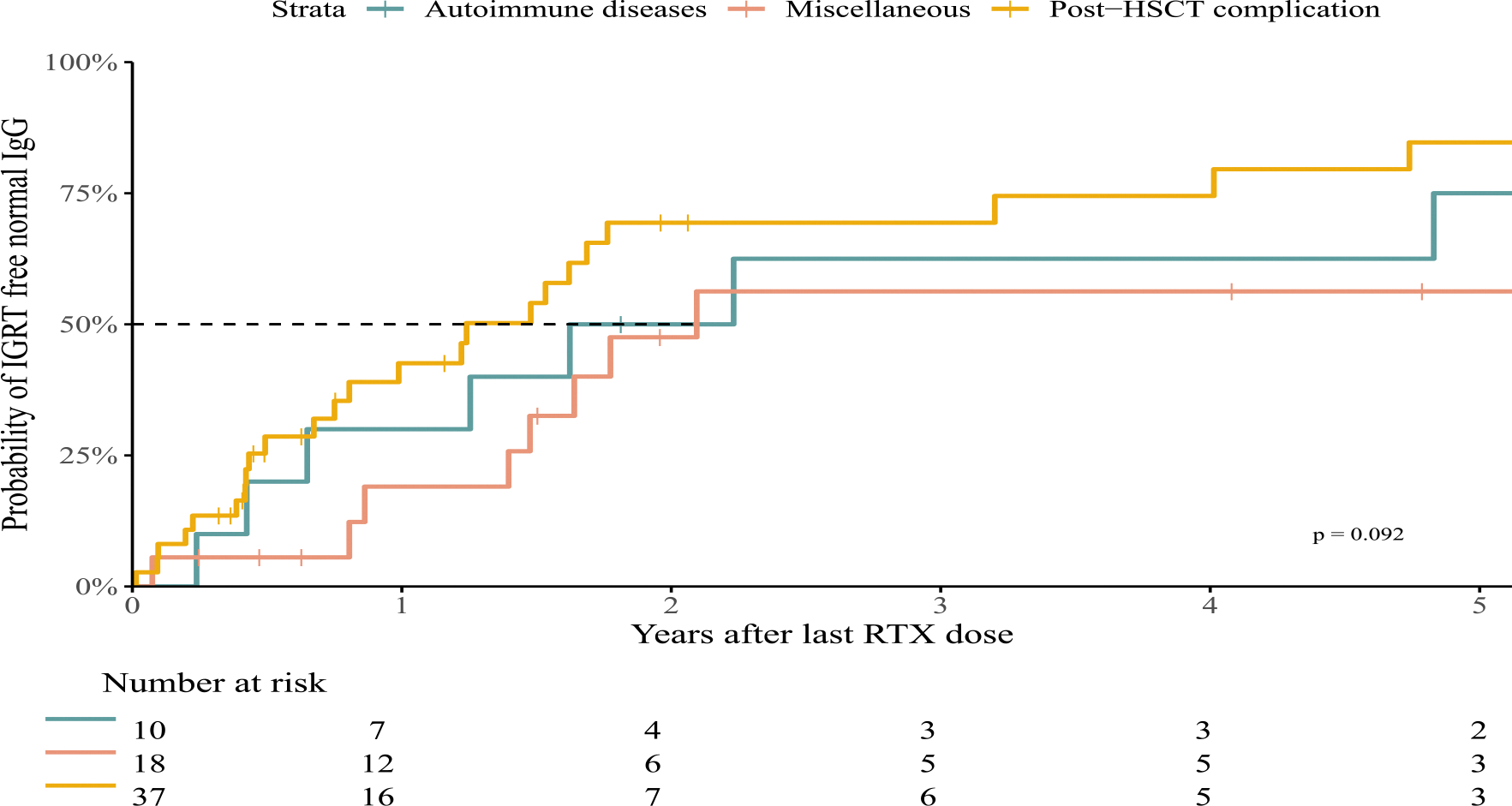
Time to IGRT-free normal IgG stratified per RTX indication. Time to IGRT-free normal IgG level after last RTX dose, in patients with HG and a minimum of 3 months of IgG follow-up measurements. Kaplan Meier curves were stratified per RTX indications: post-hematopoietic stem cell transplantation (HSCT) complication, Autoimmune diseases and a Miscellaneous group. Patients were classified as still having HG when (1) IgG levels were below −2SD compared to age matched reference values, or (2) during IGRT for the indication hypogammaglobulinemia. All groups were included in a log-rank test to test for differences (*p* = 0.092).

Recovery from hypogammaglobulinemia was assessed in 65 patients with post-RTX hypogammaglobulinemia and IgG follow-up data >3 months after the last RTX dose. Of these, 40 patients (61.5 %) demonstrated hypogammaglobulinemia recovery with a median time of 13.3 months [IQR=5.1–19.8]. Of the 25 patients with available IgG measurements >5 years after RTX therapy, 9 remained hypogammaglobulinemic (details in the “Long-term (>5 years) hypogammaglobulinemia after RTX therapy” section). Time to IGRT-free normal IgG levels did not significantly differ between RTX indications (Fig. 2, Supplementary Figure 1, *p* = 0.092). However, cox proportional-hazards analysis indicated that patients post-HSCT recovered faster than the rest of the cohort (HR=1.98, 95 %CI=[1.04–3.80], *p* = 0.037, Supplementary Figure 2).

### 3.5. B cell reconstitution

**Fig. 3.**
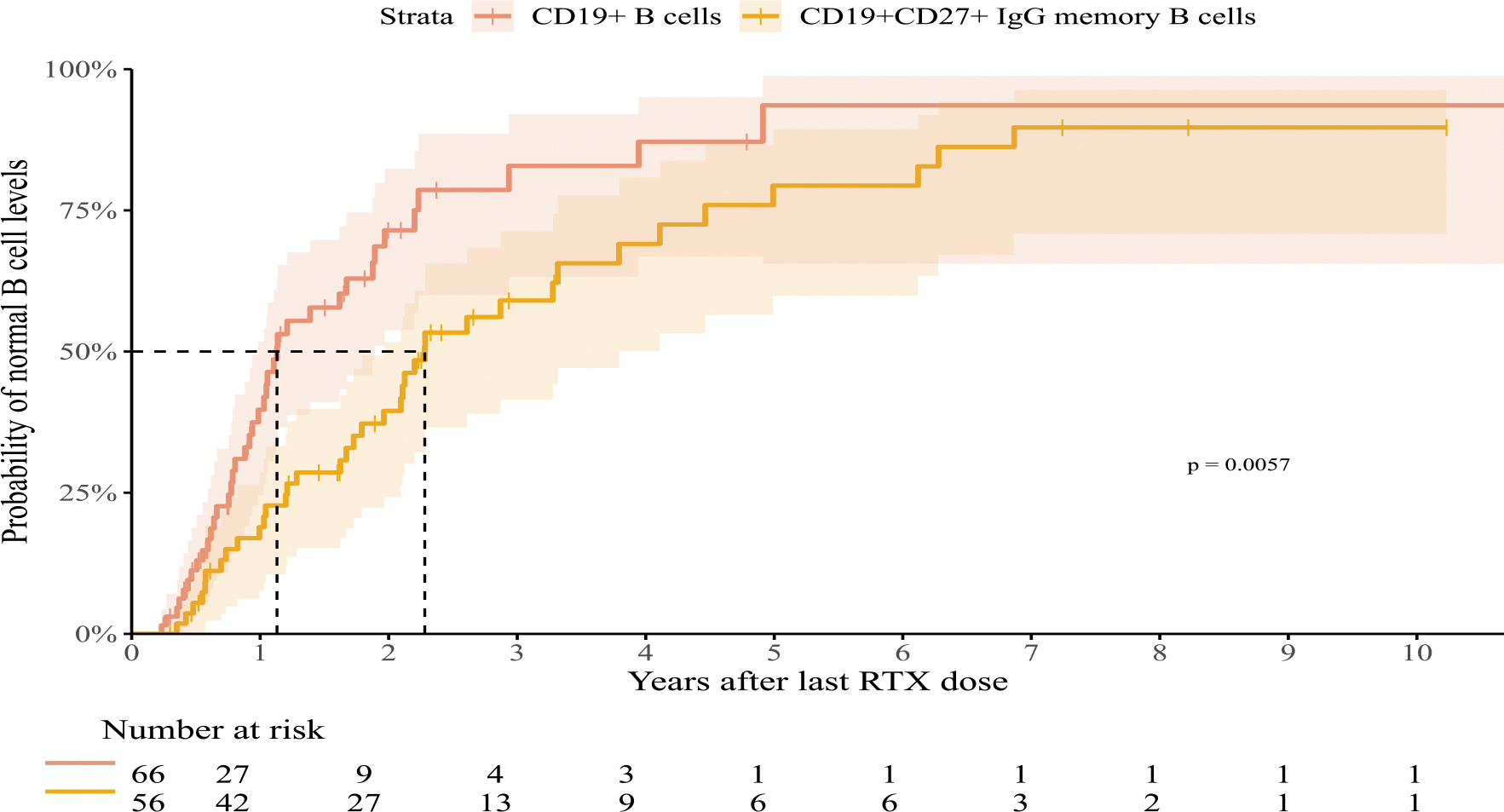
CD19+ *B* cell reconstitution compared to CD19+CD27+IgG+ switched memory B cell reconstitution. Time to CD19^+^B cell reconstitution compared to time to CD19^+^CD27^+^IgG^+^ switched memory B cell reconstitution in patients with a minimum of 3 months of B cell follow-up data after last RTX dose. Normal B cell levels were defined as measurements above −2SD from reference for age. A log-rank test was performed which showed a significant difference in Kaplan Meier curves (*p* = 0.006).

RTX led to effective peripheral B cell depletion in all patients with available measurements. Total B cell (CD19^+^) and IgG^+^ memory B cell (CD19^+^CD27^+^) reconstitution after the last dose is depicted in Fig. 3. B cell follow-up data >3 months after the last RTX dose were available in 66/134 patients (49.3 %). Median B cell follow-up time was 1.6 years (*IQR*=[0.8–3.5]). B cell reconstitution was observed in 39/66 patients (59.1 %), with a median B cell reconstitution time of 11.3 months (*IQR*= [7.3–18.0]). Among 8 patients with >5 years of follow-up, one did not achieve B cell reconstitution.

IgG^+^memory B cell follow-up data were available in 56/134 patients (41.8 %). Median follow-up time was 2.8 years (*IQR*=[1.7–5.5]). IgG^+^ memory B cell reconstitution occurred in 37/56 patients (66.1 %), at a median duration of 1.8 years (*IQR*=[1.0–2.9] years), which was significantly longer than total B cell reconstitution time (*p* = 0.006, Fig. 3). Of the 17 patients with >5 years of follow-up, 6 did not achieve IgG^+^memory B cell reconstitution 5-years post-RTX.

Post-HSCT patients exhibited faster B cell reconstitution compared to other RTX indications (*p* < 0.001, Supplementary Figure 3). Univariable Cox proportional-hazards analysis showed a 3.1 times higher likelihood of recovery in post-HSCT patients compared to those receiving RTX for other indications (HR=3.09, 95 %-CI=[1.56–6.15], *p* = 0.001). Patients who received antineoplastic agents prior to RTX therapy also exhibited faster B cell recovery (Supplementary Figure 4, HR=3.09, (95 %-CI=[1.54–6.19], *p* = 0.001)).

Older patients (4–18 years vs. 0–3 years) exhibited prolonged IgG^+^ memory B cell depletion (*p* = 0.022, Supplementary Figure 5) and a 53 % lower likelihood of recovery compared to younger patients (HR=0.47, 95 %CI=[0.24–0.91], *p* = 0.026).

No associations were observed between persistent hypogammaglobulinemia and time to B cell or IgG^+^ memory B cell reconstitution (Time to B cell reconstitution *p* = 0.45, Time to IgG memory B cell reconstitution: *p* = 0.48; Supplementary Figure 5). Patients with longer time to IgG^+^ memory B cell reconstitution, did tend to have longer hypogammaglobulinemia after RTX, but this did not reach statistical significance (HR=0.83, 95 %CI=[0.66–1.03], *p* = 0.08).

### 3.6. Long-term (>5 years) post-RTX hypogammaglobulinemia

Among the 25 patients with available IgG measurements >5 years after last RTX dose, 9 had ongoing hypogammaglobulinemia or IGRT for hypogammaglobulinemia. Characteristics of these patients are detailed in Table 3. Age at first RTX dose ranged from 1–17 years (median=10.0, IQR=[5.0–12.0]). None recovered from hypogammaglobulinemia during the study period, which extended up to 12 years (Table 3). IGRT was given to 8/9 patients post-RTX. The remaining patient did not receive IGRT despite low IgG levels, due to low infection pressure.

**Fig. 4.**
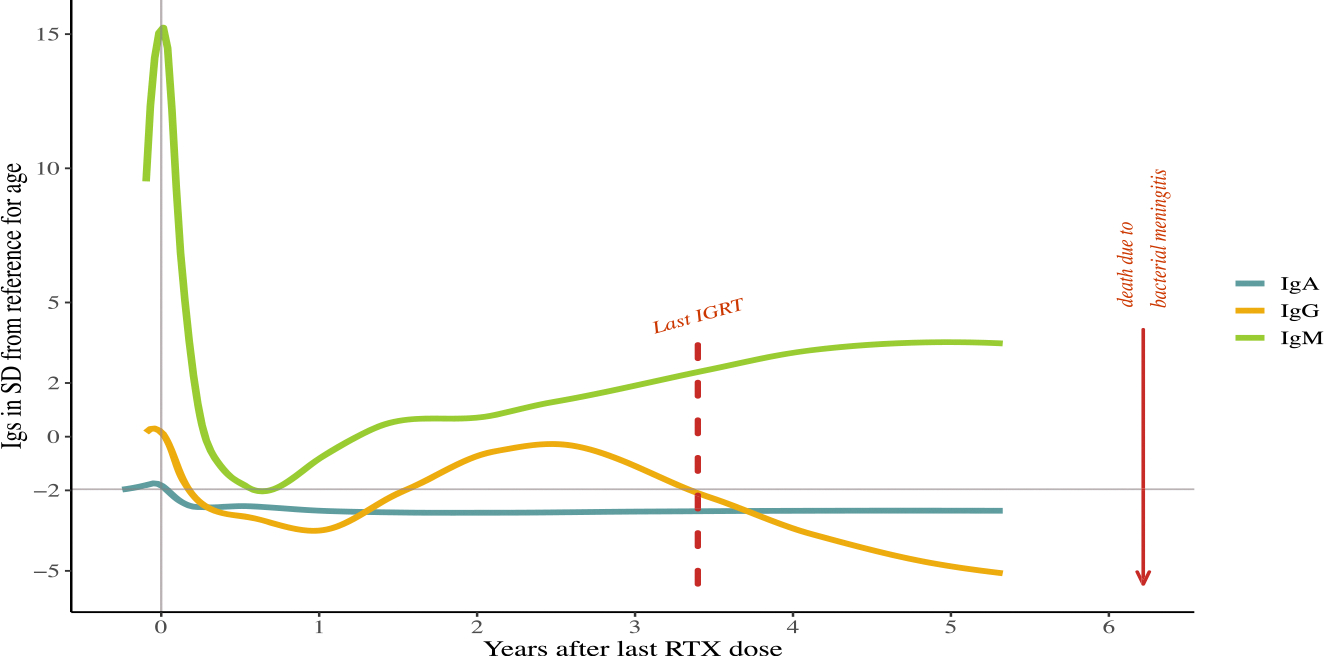
Patient with class switch recombination deficiency after RTX therapy. Ig levels (IgA, IgM, and IgG) after last RTX dose (*x* = 0) expressed in SD from reference for age. After RTX, all Ig levels dropped below reference for age on which IGRT was commenced. IGRT was halted 3 years after last RTX dose on which IgG levels dropped to −5 SD below reference for age. The patient died due to a bacterial meningitis, three years after IGRT was discontinued.

At their latest assessment (5.3–12.0 years post-RTX), 5/9 patients showed decreased levels of IgG^+^ memory B cells. These patients all had either hypo-IgM and/or hypo-IgA in addition to their hypo-IgG, with 2/5 showing hyper-IgM in conjunction with hypo-IgA (see Table 3). Only one patient had a pre-existing PID diagnosis before RTX therapy, with no defects found in PID genetic testing. Three more patients underwent post-RTX genetic testing for PID, but no defects were identified. Three patients were diagnosed with a class-switch recombination-deficiency after RTX therapy (patients 4, 5, and 9, Table 3). Fig. 4 illustrates longitudinal Ig levels in one of the patients with a post-RTX class-switch recombination deficiency. This patient received RTX following HSCT and demonstrated complete reconstitution of CD19^+^ *B* cells and T cells. However, IgG levels remained depleted, despite the absence of immunosuppressive medication. The patient died of a bacterial meningitis 3 years after IGRT was discontinued per patient’s wish.

### 3.7. Recurrent infections

Recurrent infections after RTX therapy were reported in 18/134 patients (13.4 %). Of these, seventeen had post-RTX hypogammaglobulinemia, and of the 12 patients with available IgG measurements >6 months post-RTX, 10 had persistent HG. Patients with persistent HG had 6.17 higher odds of developing recurrent infections (95 %-CI=[1.20–61.58], *p* = 0.014). RTX indications of children with recurrent infections included post-HSCT complications (*n* = 9), autoimmune diseases (*n* = 2) and miscellaneous (*n* = 7). Sixteen out of 18 patients with recurrent infections received IGRT. In 7 patients with recurrent infections, these could be (at least partly) attributed to RTX-related hypogammaglobulinemia, as these were new-onset bacterial sinopulmonary infections during hypogammaglobulinemia. During follow-up, 21 patients died, with 2 deaths due to or complicated by proven bacterial infection during persistent hypogammaglobulinemia. Both patients had low IgG levels, and one was not receiving IGRT at the time of death.

## 4. Discussion

This study assessed prevalence, clinical characteristics and outcomes of hypogammaglobulinemia after RTX therapy in a heterogenous group of pediatric patients including the largest post-HSCT cohort reported to date. Hypogammaglobulinemia after RTX therapy was highly prevalent with low IgG levels and/or IGRT for hypogammaglobulinemia observed in 55 %. Of patients with normal IgG levels before RTX therapy, over half developed new onset hypogammaglobulinemia. Hypogammaglobulinemia frequently persisted beyond 6 months, often lasting for years and necessitating long-term IGRT. Additionally, persistent hypogammaglobulinemia occurred in some patients despite numerical immune cell reconstitution, also in the post-HSCT setting. Recurrent infections were recorded in 18 patients and were more prevalent in children with persistent hypogammaglobulinemia. Two infections proved fatal. These findings underscore the clinical relevance of post-RTX hypogammaglobulinemia in this population and highlight the need for careful monitoring.

The total B cell and IgG^+^switched memory B cell reconstitution periods observed in this study are in line with previous reports [11,20, 31–35]. Contrary to some reports, no association was found between CD19^+^ *B* cell reconstitution and hypogammaglobulinemia recovery post-RTX, even though depletion of naïve B cells as progenitors of plasma cells may lead to hypogammaglobulinemia [11,25,36,37]. Whilst not statistically significant, a tendency was seen between delayed IgG^+^ memory B cell recovery and hypogammaglobulinemia in this study. Moreover, over half of the group of patients with hypogammaglobulinemia persisting for >5 years had depleted levels of IgG memory B cells at latest assessment, whereas all had normal peripheral total B cells counts. Several case reports and series have previously speculated on this link between switched memory B cells and Ig production post-RTX, suggesting impaired B cell maturation to be a possible cause of persistent hypogammaglobulinemia [11,34,36–41]. A decreased fraction of IgG^+^ memory B cells could therefore potentially serve as a biomarker for identifying patients requiring long-term IGRT, as suggested in earlier literature [42]. Another possible manifestation of impaired B cell maturation post-RTX is the class-switch recombination-deficiencies we observed in three cases. This phenomenon was only recently linked to RTX and was thus far considered to be profoundly rare [11,35,38,43,44]. Alternatively, in the largest pediatric cohort study on post-RTX hypogammaglobulinemia to date by Labrosse et al., comparable patients with post-RTX hypogammaglobulinemia were classified as primary immune deficiencies unveiled by RTX. However, a more recent study by De Bruin et al. showed that an impaired IgG^+^ memory B cell maturation post-RTX could not be attributed to intrinsic B cell defects, as in vitro stimulation of these cells led to normal maturation and class-switch recombination abilities [41]. Further research is warranted to define the role of B cell maturation defects in post-RTX hypogammaglobulinemia.

Considering that previous studies investigating hypogammaglobulinemia after pediatric HSCT reported rates as high as 70–77 %, [45,46] it is unsurprising that post-HSCT patients exhibited a similarly high prevalence of hypogammaglobulinemia (67.2 %) and persistent hypogammaglobulinemia (52.4 %) in this study. While the intensive immune-modulating/-depleting regimens these patients undergo before RTX therapy likely contribute to their persistent hypogammaglobulinemia status, we found no association between immunomodulating medications (other than RTX) and persistent hypogammaglobulinemia. These findings align with the study by Labrosse et al. [23]. Nonetheless, as we only investigated broad classifications of immunosuppressive medications, we may have missed negative associations with specific medications as reported in other studies (e.g. pulse corticosteroids, anti-metabolites, cyclophosphamide, and mycophenolate mofetil) [11, 14,16,21]. Notably, post-HSCT patients, despite their high prevalence of persistent hypogammaglobulinemia, demonstrated faster reconstitution of hypogammaglobulinemia, B cells and IgG memory B cells compared to other indications. This faster immune recovery may be due to reconstitution from healthy donor stem cells, as compared to immune recovery from intrinsically affected B cells. Alternatively, the fact that B cell measurements were conducted more frequently in post-HSCT patients could have facilitated earlier detection of reconstitution. Younger age (0–3 years) was also associated with faster IgG memory B cell recovery, with no correlation found between age and persistent hypogammaglobulinemia. The latter contrasts with several smaller pediatric studies reporting younger age to be a predictor of (persistent) hypogammaglobulinemia, [20,21,33,47] but again is in line with findings reported by Labrosse et al. [23]. In accordance with pediatric and adult literature [11,19,23,26], low IgG and IgM levels pre-RTX were associated with persistent hypogammaglobulinemia, thereby stressing the importance of determining Ig levels prior to RTX therapy. This study also reinforces prior evidence associating hypogammaglobulinemia after RTX therapy to recurrent infections in pediatric patients [11–14,21,23, 24].

Overall, our reported rates of hypogammaglobulinemia and persistent hypogammaglobulinemia in children are higher than previous reports in both children and adults [13–24,26]. This may be due to the wide variety of included conditions and/or differences in definitions of (persistent) hypogammaglobulinemia. While some studies defined persistent hypogammaglobulinemia as >1 year after first RTX dose, others, including us, defined it as >6 months after the last RTX dose. In addition, our classification of hypogammaglobulinemia included patients receiving IGRT for hypogammaglobulinemia, rather than only IgG levels <−2*SD*. This broader classification allows for the inclusion of patients who require IGRT due to severe or prolonged hypogammaglobulinemia, thereby reducing potential selection bias and providing real-world data representative of daily clinical practice. Moreover, our high hypogammaglobulinemia occurrence may be due to the substantial proportion of post-HSCT patients within our cohort [45,46]. Children receiving RTX for autoimmune disorders typically undergo fewer additional immune-depleting therapies than HSCT patients. Thus, the prevalence of hypogammaglobulinemia in this group (24 %) may better reflect the direct effect of RTX on IgG levels and aligns with previous reports [15,16,19–21,23].

This study has its limitations due to the retrospective design. As previously mentioned, IgG levels and B cell numbers were more frequently measured in certain subgroups, which could have led to earlier detection of hypogammaglobulinemia and B cell recovery. Frequent loss to follow-up and the need to combine clinically heterogeneous RTX indications limited the study’s power for multivariate and subgroup analyses. Furthermore, as IgG levels may have been monitored more closely or longer in patients with longer or more severe hypogammaglobulinemia or recurrent infections, this could have resulted in an overestimation of (persistent) hypogammaglobulinemia rates. Conversely, we observed that laboratory assessments of IgG were regularly stopped despite patients still having hypogammaglobulinemia, possibly leading to underestimation of hypogammaglobulinemia. Lastly, 11 % of patients with post-RTX hypogammaglobulinemia had severe intestinal GvHD for at least a period during follow-up, possibly leading to hypogammaglobulinemia through protein loss and an overestimation of RTX-induced hypogammaglobulinemia.

In conclusion, around 50 % of children develop (persistent) hypogammaglobulinemia after RTX therapy. Children who develop hypogammaglobulinemia regularly need IGRT and are significantly more likely to suffer from recurrent infections, which can be fatal. Clinicians should be aware that patients frequently develop severe or prolonged hypogammaglobulinemia post-RTX, occasionally persisting over 5 years. Secondary class-switch recombination-deficiency should be considered in such cases. Prospective studies are needed to confirm these findings. We recommend determining IgG levels before start of RTX in all patients and close monitoring after, especially in those with low IgG levels pre-RTX. Monitoring should not cease until normal IgG levels are observed, regardless of total B cell reconstitution.

## Supplementary Material

MMC1

Supplementary material associated with this article can be found, in the online version, at doi:10.1016/j.clicom.2025.04.001.

## Figures and Tables

**Table 1:** Baseline characteristics.

	Post-HSCT complication	Autoimmune Diseases	Miscellaneous	P*

Number of patients	64	42	28	
Female sex (n (%))	27 (42.2)	26 (61.9)	7 (25.0)	0.008
Mean age in years (*SD*)	6.94 (5.98)	11.48 (4.92)	11.89 (4.42)	<0.001
*Baseline Igs and B cell counts* **				
IgG (median *(IQR) [SD]*)	−1.40 [−1.87, −0.47]	− 0.40 [−1.58, 1.57]	−1.71 [−2.20, − 0.91]	0.006
IgA (median *(IQR) [SD]*)	−1.60 [−2.15, − 0.94]	−1.00 [−1.84, −0.62]	−1.69 [−1.89, − 0.71]	0.343
IgM (median *(IQR) [SD]*)	−1.09 [−1.73, 0.95]	− 0.54 [−1.25, 0.18]	−1.23 [−1.96, − 0.17]	0.365
CD19^+^ *B* cell counts (median *(IQR) [SD]*)	− 2.15 [−3.15, − 0.49]	− 0.37 [−1.67, 1.69]	−1.77 [−2.81, 0.22]	0.088
CD19^+^CD27^+^ IgG^+^ memory *B* cell counts (median *(IQR) [SD]*)	−1.70 [−2.58, −0.57]	1.06 [−0.50, 1.23]	− 0.94 [−1.69, − 0.21]	0.011
*RTX therapy regimen*				
Number of RTX cycles (median [min, max]) ^a^	1 [1, 4]	1 [1, 6]	1 [1, 3]	0.321
Number of RTX doses (median [min, max])	3 [1, 12]	4 [2, 15]	4 [1, 10]	0.003
Relative cumulative dose in mg/m2 (median [IQR])	1140 [761, 1432]	1390 [1116, 1508]	1521 [748, 2207]	0.023
*Medication prior to RTX therapy regimen* ^b^				
Corticosteroids (n (%)) ^c^	58 (90.6)	30 (71.4)	23 (82.1)	0.037
Antineoplastic agents (n (%)) ^d^	54 (84.4)	3 (7.1)	12 (42.9)	<0.001
Immunosuppressants or immunomodulators (n (%)) ^e^	59 (92.2)	30 (71.4)	23 (82.1)	0.018
(Other) biological DMARDs (n (%)) ^f^	28 (43.8)	3 (7.1)	4 (14.3)	<0.001
IGRT within five months prior to RTX (n (%))	35 (54.7)	10 (23.8)	5 (17.9)	<0.001
*Medication after RTX therapy commencement* ^b^				
Corticosteroids (n (%)) ^c^	63 (98.4)	38 (90.5)	25 (89.3)	0.118
Antineoplastic agents (n (%)) ^d^	26 (40.6)	5 (11.9)	14 (50.0)	0.001
Immunosuppressants or immunomodulators (n (%)) ^e^	63 (98.4)	38 (90.5)	26 (92.9)	0.173
(Other) biological DMARDs (n (%)) ^f^	13 (20.3)	4 (9.5)	2 (7.1)	0.145
IGRT during or after RTX therapy (n (%))	56 (87.5)	18 (42.9)	11 (39.3)	<0.001
Pre-RTX hypogammaglobulinemia (n/total available (%))	14/61 (23.0)	5/32 (15.6)	10/22 (45.5)	0.039

* P-values were calculated using Chi-squared test for categorical and one-way ANOVA for continuous veriables; Kruskal-Wallis and Fisher test were used for nonnormally distributed and low (expected) count variables, respectively.

** Ig and B cell levels are expressed in SD from reference for age.

a A cycle was defined as one or more RTX doses with no >45 days between courses.

b Medication was classified according to the ATC/DDD Index 2021 [30].

c Prednisone, dexamethasone, triamcinolone, hydrocortisone.

d Cyclophosphamide and other alkylating agents, methotrexate and other antimetabolites, protein kinase inhibitors, miscellaneous.

e Mycophenolic acid, sirolimus, leflunomide, everolimus, cyclosporine, tacrolimus, azathioprine, methotrexate.

f Thymoglobulin, abatacept, infliximab, adalimumab, tocilizumab, miscellaneous.

**Table 2:** Hypogammaglobulinemia (HG) pre- and post-RTX by RTX indication.

	Pre-RTX HG *Low IgG* (n/total available (%))	Post-RTX HG^a^ *Low IgG or receiving IGRT* (n/total available (%))	Persistent HG^b^ *Low IgG or receiving IGRT (>6 months after RTX)* (n/total available (%))	p-value**

Post-HSCT complication	14/61 (23.0)	43/64 (67.2)	22/42 (52.4)	<0.001
Autoimmune diseases	5/32 (15.6)	10/42 (23.8)	8/29 (27.6)	0.450
Miscellaneous	10/22 (45.5)	21/28 (75.0)	15/20 (75.0)	0.046
Overall	29/115 (25.2)	74/134 (55.2)	45/91 (49.5)	<0.001
p-value*	0.039	<0.001	0.004	

* Differences in occurrence of hypogammaglobulinemia between RTX indications, calculated using the Fisher exact test.

** Differences in hypogammaglobulinemia rates pre- and post-RTX, calculated using the McNemar test.

a In 3/74 (4.1 %) patients with post-RTX hypogammaglobulinemia, classification of post-RTX hypogammaglobulinemia was solely based on IGRT, without low IgG levels measured after last RTX dose. In 2/3 of these patients, however, low IgG levels were measured before last RTX dose.

b In 4/45 (8.9 %) patients with persistent hypogammaglobulinemia, no low IgG levels were reported after 6 months of follow-up, and classification was solely based on IGRT after 6 months. In 3 of these patients, low IgG levels had been measured within the first 6 months follow-up.

**Table 3:** Characteristics of patients with HG >5 years post-RTX.

RTX indication		HG pre-RTX? *(SD from ref. for age)*	Follow-up after last RTX dose in years	Igs at last measurement^a^			B cells at last measurement^a^		IGRT at last IgG measurement?^b^	IGRT indication as reported by treating physician	PID genepanel tested?^c^

				IgA in g/L *(ref. for age)*	IgM in g/L *(ref. for age)*	IgG in g/L *(ref. for age)*	CD19^+^ *B* cells *(ref. for age)*	CD19^+^CD27^+^IgG^+^ switched memory B cells *(ref. for age)*			
1	Autoimmune disease	No (−1.66)	10.49	3.20 (0.7–4.0)	1.00 (0.4–2.3)	6.91 (7–16)	143 (114–436)	2.2 (0.8–11.7)	Yes	HG/treatment autoimmune disorder	Yes (negative)
2	Autoimmune disease	No (−1.52)	9.83	1.20 (0.62–3.0)	0.57 (0.13–1.6)	10.8 (5.2–15.6)	1114 (116–555)	1.8 (1.5–8.8)	Yes	HG	Yes (negative)
3	Hematological malignancy	Yes (− 3.0)	6.80	0.16 (0.7–4.0)	0.26 (0.4–2.3)	4.55 (7–16)	NA	NA	No	HG	No
4	Immunodeficiency	No (−0.41)	11.98	0.64 (0.7–4.0)	2.00 (0.4–2.3)	0.70 (7–16)	311 (114–436)	0.5 (0.8–11.7)	No	HG	Yes (negative)
5	Nephrotic Syndrome	NA	8.23	0.04 (0.7–3.6)	10.0 (0.28–2.4)	0.20 (5.2–15.6)	133 (119–578)	0.7 (2.1–9.4)	No	NA	Yes (negative)
6	Post-HSCT complication	No (−0.57)	5.61	1.80 (0.7–4.0)	1.00 (0.4–2.3)	5.40 (7–16)	651 (114–436)	1.6 (0.8–11.7)	No	HG (but previously given for autoimmune cytopenias)	No
7	Post-HSCT complication	No (−1.86)	8.56	0.07 (0.7–3.6)	0.08 (0.28–2.4)	7.87 (5.2–15.6)	159 (119–578)	0.2 (2.1–9.4)	Yes	HG (but previously given for autoimmune cytopenias)	No
8	Post-HSCT complication	No (0.34)	6.07	0.03 (0.7–4.0)	0.02 (0.4–2.3)	10.80 (7–16)	404 (114–436)	0.0 (0.8–11.7)	Yes	HG	No
9	Post-HSCT complication	No (−0.93)	5.33	0.03 (0.7–4.0)	2.80 (0.4–2.3)	0.60 (7–16)	645 (114–436)	0.3 (0.8–11.7)	No	HG	No

Bold indicates below or above reference for age. ref. indicates reference values; NA, not available.

a Measured in the peripheral blood.

b (Within) 5 months prior to IgG measurement.

c Gene panel including CD40L/CD40-, AID, UNG, NEMO, IkBα, ATM, and PMS2.

## Data Availability

Data will be made available upon reasonable request.

## Data availability

Data will be made available on request.

## References

[1] Boross P, Leusen JH, Am. J. Cancer Res. 2 (6) (2012) 676–690. 23226614 PMC3512181

[2] Cragg MS, Walshe CA, Ivanov AO, Glennie MJ, Curr. Dir. Autoimmun. 8 (2005) 140–174, 10.1159/000082102. 15564720

[3] Pavlasova G, Mraz M, Haematologica 105 (6) (2020) 1494–1506, 10.3324/haematol.2019.243543. 32482755 PMC7271567

[4] Barcellini W, Fattizzo B, Zaninoni A, Blood Med J. 10 (2019) 265–278, 10.2147/JBM.S190327. Published 2019 Aug 8. PMC6690850 31496855

[5] Lindsay J, Yong MK, Greenwood M, , Rev. Med. Virol. 30 (4) (2020) e2108, 10.1002/rmv.2108. 32301566

[6] Zian Z, Berry SPD, Bahmaie N, , Int. Immunopharmacol. 95 (2021) 107565, 10.1016/j.intimp.2021.107565. 33773205

[7] Moradzadeh M, Aghaei M, Mehrbakhsh Z, Arab-Bafrani Z, Abdollahi N, Clin. Rheumatol. 40 (10) (2021) 3897–3918, 10.1007/s10067-021-05698-4. 33796953

[8] Girard J, Karimi Y, Carty S, , Curr. Hematol. Malig. Rep. 16 (1) (2021) 25–31, 10.1007/s11899-021-00614-8. 33754292

[9] Iijima K, Sako M, Nozu K, Clin. Exp. Nephrol. 21 (2) (2017) 193–202, 10.1007/s10157-016-1313-5. 27422620 PMC5388729

[10] Salles G, Barrett M, Foà R, , Adv. Ther. 34 (10) (2017) 2232–2273, 10.1007/s12325-017-0612-x. PMC5656728 28983798

[11] Sacco KA, Abraham RS, Immunotherapy. 10 (8) (2018) 713–728, 10.2217/imt-2017-0178. 29569510

[12] Casulo C, Maragulia J, Zelenetz AD, Clin. Lymphoma Myeloma Leuk. 13 (2) (2013) 106–111, 10.1016/j.clml.2012.11.011. 23276889 PMC4035033

[13] Barmettler S, Ong MS, Farmer JR, Choi H, Walter J, JAMa Netw. Open. 1 (7) (2018) e184169, 10.1001/jamanetworkopen.2018.4169. Published 2018 Nov 2. 30646343 PMC6324375

[14] Christou EAA, Giardino G, Worth A, Ladomenou F, Int. Rev. Immunol. 36 (6) (2017) 352–359, 10.1080/08830185.2017.1346092. 28800262

[15] Khojah AM, Miller ML, Klein-Gitelman MS, , Pediatr. Rheumatol. Online J. 17 (1) (2019) 61, 10.1186/s12969-019-0365-y. Published 2019 Aug 28. 31462263 PMC6712749

[16] Newman EN, Israelsen RB, Williamson K, Hsieh EWY, Ann. Allergy Asthma Immunol. 128 (2) (2022) 225–226, 10.1016/j.anai.2021.10.028. 34728346

[17] Fujinaga S, Ozawa K, Sakuraya K, Yamada A, Shimizu T, Clin. Nephrol. 85 (6) (2016) 340–345, 10.5414/CN108835. 27125626

[18] Fujinaga S, Nishino T, Umeda C, Tomii Y, Watanabe Y, Sakuraya K, Pediatr. Nephrol. 34 (2) (2019) 353–357, 10.1007/s00467-018-4145-6. 30426219

[19] Inoki Y, Kamei K, Nishi K, Sato M, Ogura M, Ishiguro A, Pediatr. Nephrol. 37 (5) (2022) 1057–1066, 10.1007/s00467-021-05304-4. 34606002

[20] Wennmann M, Kathemann S, Kampmann K, , Front. Pediatr. 9 (2021) 651323, 10.3389/fped.2021.651323. Published 2021 Nov 30. 34917554 PMC8669827

[21] Ong MS, Rothman D, Barmettler S, , Rheumatology. (Oxford) 61 (4) (2022) 1610–1620, 10.1093/rheumatology/keab626. 34329428

[22] Ottaviano G, Marinoni M, Graziani S, , J. Allergy Clin. Immunol. Pract. 8 (1) (2020) 273–282, 10.1016/j.jaip.2019.07.032. 31377437

[23] Labrosse R, Barmettler S, Derfalvi B, , J. Allergy Clin. Immunol. 148 (2) (2021) 523–532, 10.1016/j.jaci.2021.03.041, e8. 33862010

[24] McAtee CL, Lubega J, Underbrink K, , JAMa Netw. Open. 4 (2) (2021) e2036321, 10.1001/jamanetworkopen.2020.36321. Published 2021 Feb 1. 33533931 PMC7859842

[25] Simon AK, Hollander GA, McMichael A, Proc. Biol. Sci. 282 (1821) (2015) 20143085, 10.1098/rspb.2014.3085. 26702035 PMC4707740

[26] Boleto G, Avouac J, Wipff J, , Semin. Arthritis Rheum. 48 (2) (2018) 149–154, 10.1016/j.semarthrit.2018.02.010. 29548542

[27] ten Berg MJ, Huisman A, van den Bemt PM, Schobben AF, Egberts AC, van Solinge WW, Clin. Chem. Lab. Med. 45 (1) (2007) 13–19, 10.1515/CCLM.2007.009. 17243908

[28] Author unknown. IgA, IgG, IgM kwantitatief + high resolution elektroforese. In: Products and Services, Diagnostic Tests. Sanquin Blood Supply Foundation. https://www.sanquin.org/nl/producten-en-diensten/diagnostiek/diagnostische-testen/index/name/s901-iga-igg-igm-kwantitatief-high-resolution-elektroforese. Accessed 15 oct 2021.

[29] Author unknown. IgG memory B cells. In: Laboratory Assessments. Central Diagnostic Laboratory University Medical Centre Utrecht: “What’s Lab”. http://lkch.nl/bepalingen/Front/Details/3254. Accessed 15 oct 2021.

[30] WHO Collaborating Centre for Drug Statistics Methodology, ATC Classification Index with DDDs, 2021. Oslo, Norway 2020.

[31] Worch J, Makarova O, Burkhardt B, Cancers. (Basel) 7 (1) (2015) 305–328, 10.3390/cancers7010305. Published 2015 Jan 29. 25643241 PMC4381260

[32] Mitchell C, Crayne CB, ACR. Open. Rheumatol. 1 (8) (2019) 527–532, 10.1002/acr2.11074. Published 2019 Aug 27. 31777835 PMC6858005

[33] Colucci M, Carsetti R, Serafinelli J, , Front. Immunol. 10 (2019) 1653, 10.3389/fimmu.2019.01653. Published 2019 Jul 16. 31379849 PMC6646679

[34] Nishio M, Fujimoto K, Yamamoto S, , Br. J. Haematol. 137 (4) (2007) 349–354, 10.1111/j.1365-2141.2007.06584.x. 17456057

[35] Kaplan B, Kopyltsova Y, Khokhar A, Lam F, Bonagura V, Allergy Clin J. Immunol. Pract. 2 (5) (2014) 594–600, 10.1016/j.jaip.2014.06.003. 25213054

[36] Ottaviano G, Sgrulletti M, Moschese V, Eur. J. Immunol. 52 (10) (2022) 1572–1580, 10.1002/eji.202149667. 35892275

[37] Thiel J, Rizzi M, Engesser M, , Arthritis Res. Ther. 19 (1) (2017) 101, 10.1186/s13075-017-1306-0. Published 2017 May 18. 28521808 PMC5437549

[38] Mizuhara K, Fujii N, Meguri Y, , Int. J. Hematol. 112 (3) (2020) 422–426, 10.1007/s12185-020-02886-x. 32342335

[39] Nishio M, Fujimoto K, Yamamoto S, , Eur. J. Haematol. 77 (3) (2006) 226–232, 10.1111/j.1600-0609.2006.00693.x. 16923109

[40] Irie E, Shirota Y, Suzuki C, , Int. J. Hematol. 91 (3) (2010) 501–508, 10.1007/s12185-010-0528-6. 20217285

[41] Ott de Bruin LM, Pico-Knijnenburg I, van Ostaijen-Ten Dam MM, , Int. J. Mol. Sci. 24 (21) (2023) 16012, 10.3390/ijms242116012. Published 2023 Nov 6. 37958995 PMC10649739

[42] Marzollo A, Serena T, Mainardi C, , J. Pediatr. Hematol. Oncol. 45 (1) (2023) e145–e149, 10.1097/MPH.0000000000002582. 36598967

[43] Luterbacher F, Bernard F, Baleydier F, Ranza E, Jandus P, Blanchard-Rohner G Case, Front. Immunol. 12 (2021) 773853, 10.3389/fimmu.2021.773853. Published 2021 Dec 22. 35003091 PMC8727997

[44] Clark R, Lindsey D, Whiteway S, Mikita C, Lieuw K, J. Pediatr. Hematol. Oncol. 43 (4) (2021) e601–e604, 10.1097/MPH.0000000000001871. 32590421

[45] Lim SH, Zhang Y, Wang Z, , Bone Marrow Transplant. 35 (2) (2005) 207–208, 10.1038/sj.bmt.1704742. 15531902

[46] Frangoul H, Min E, Wang W, , Bone Marrow Transplant 48 (11) (2013) 1456–1459, 10.1038/bmt.2013.76. 23708706

[47] Sinha A, Mathew G, Arushi A, , Nephrol. Dial. Transplant. 38 (4) (2023) 939–949, 10.1093/ndt/gfac228. 36071552

