# Supplementary material for: Persistent hypogammaglobulinemia after rituximab therapy in pediatric patients, prevalence and clinical outcomes
Source: Clin Immunol Commun. Author manuscript; Available in PMC 2026 Apr 8. (PMC13056382; doi:10.1016/j.clicom.2025.04.001)
Supplement: MMC1 [file NIHMS2148895-supplement-MMC1.docx]

# Supplementary Material

**Supplementary figures**

**Supplementary Figure 1. Time to IGRT-free normal IgG stratified RTX indications grouped under Miscellaneous.** Time to IGRT-free normal IgG level after last RTX dose in patients with hypogammaglobulinemia and a minimum of 3 months of IgG follow-up measurements. Patients were classified as still having hypogammaglobulinemia when (1) IgG levels were below -2SD compared to age matched reference values, or (2) during IGRT for indication hypogammaglobulinemia. A log-rank test was performed, which did not show a significant difference in Kaplan Meier curves between indications (p=0.37).

**Supplementary Figure 2. Time to IGRT-free normal IgG stratified RTX indication.** Time to IGRT-free normal IgG level after last RTX dose in patients with hypogammaglobulinemia and a minimum of 3 months of IgG follow-up measurements. Kaplan Meier curves are stratified per history of hematopoietic stem cell transplantation (HSCT) prior to RTX therapy. Patients were classified as still having hypogammaglobulinemia when (1) IgG levels were below -2SD compared to age matched reference values, or (2) during IGRT for the indication hypogammaglobulinemia. A log-rank test was performed, which showed a significant difference in Kaplan Meier curves (p=0.034).

**Supplementary Figure 3. CD19^+^ B cell reconstitution stratified by RTX indication.** CD19^+^ B cell reconstitution after last RTX dose in patients receiving RTX therapy for post-HSCT complications compared to other indications. Normal B cell levels were defined as measurements above -2SD from reference for age. A log-rank test was performed, which showed a significant difference in Kaplan Meier curves (p<0.001).

**Supplementary Figure 4. CD19^+^ B cell reconstitution stratified by antineoplastic agent exposure prior to RTX therapy.** CD19^+^ B cell reconstitution after last RTX dose in patients having received antineoplastic agent prior to receiving RTX therapy compared to patient unexposed to antineoplastic agents prior to RTX. A minimum of 3 months of CD19^+^ B cell follow-up measurements was implemented. Normal B cell levels were defined as measurements above -2SD from reference for age. A log-rank test was performed, which showed a significant difference in Kaplan Meier curves (p<0.001).

**Supplementary Figure 5. CD19^+^CD27^+^IgG^+^ switched memory B cell reconstitution stratified by age group.** CD19^+^CD27^+^IgG^+^ memory B cell reconstitution after last RTX dose in patients aged 0-3 years compared to aged 4-18 years. A minimum of 3 months of CD19^+^CD27^+^IgG^+^ memory B cell follow-up measurements was implemented. Normal B cell levels were defined as measurements above -2SD from reference for age. A log-rank test was performed, which showed a significant difference in Kaplan Meier curves (p=0.022).

**Supplementary Figure 6. CD19^+^ B cell and CD19^+^CD27^+^IgG^+^ switched memory B cell reconstitution stratified by persistent hypogammaglobulinemia.** (A) CD19^+^ B cell reconstitution and (B) CD19^+^CD27^+^IgG^+^ memory B cell reconstitution after last RTX dose in patients with persistent hypogammaglobulinemia (>6 months) compared to patients with transient (<6 months) or no hypogammaglobulinemia after last RTX dose. A minimum of 3 months follow-up measurements was implemented in both panels shown. Normal B cell levels were defined as measurements above -2SD from reference for age. Log rank tests found no association between persistent hypogammaglobulinemia and (A) CD 19^+^ B cell reconstitution times (p=0.36); and/or (B) IgG^+^ memory B cell reconstitution times (p=0.58).

| RTX indication |  | Autoimmune diseases | Immunodeficiencies | Hematological malignancies | Nephrotic syndrome | Post-HSCT complication | Post-solid organ transplantation complication | **Overall** | P* |
| --- | --- | --- | --- | --- | --- | --- | --- | --- | --- |
| Number of patients |  | 42 | 4 | 13 | 5 | 64 | 6 | **134** |  |
| Female sex (n (%)) |  | 26 (61.9) | 1 (25.0) | 4 (30.8) | 1 (20.0) | 27 (42.2) | 1 (16.7) | **60 (44.8)** | 0.076 |
| Age in years (mean ±*SD*) |  | 11.5 (4.92) | 9.75 (6.40) | 12.0 (4.12) | 12.00 (4.36) | 6.94 (5.98) | 13.0 (4.43) | **9.40 (5.83)** | **<0.001** |
| *Baseline immunoglobulins and B cell count* |  |  |  |  |  |  |  |  |  |
| IgG (median (IQR) [SD]) |  | -0.40 [-1.58, 1.57] | -0.96 [-1.24, -0.69] | -1.96 [-2.59, -1.60] | -2.13 [-2.44, -1.67] | -1.40 [-1.87, -0.47] | -0.87 [-1.74, 0.58] | **-1.14 [-1.92, -0.31]** | **0.011** |
| IgA (median (IQR) [SD]) |  | -1.00 [-1.84, -0.62] | -1.60 [-2.01, -1.23] | -1.83 [-2.13, -1.65] | -1.17 [-1.62, -0.60] | -1.60 [-2.15, -0.94] | -1.31 [-1.76, -0.62] | **-1.54 [-2.04, -0.71]** | 0.437 |
| IgM (median (IQR) [SD]) |  | -0.54 [-1.25, 0.18] | -1.66 [-2.17, 20.11] | -1.69 [-1.98, -0.11] | -1.03 [-1.21, -0.16] | -1.09 [-1.73, 0.95] | -1.03 [-1.16, -0.26] | **-0.93 [-1.71, 0.33]** | 0.676 |
| CD19^+^ B cell counts (median (IQR) [SD]) |  | -0.37 [-1.67, 1.69] | -3.24 [-3.29, -3.20] | NA [NA, NA] | 1.02 [0.48, 3.38] | -2.15 [-3.15, -0.49] | -1.98 [-2.34, -1.77] | **-1.73 [-2.97, 0.13]** | **0.030** |
| CD19^+^CD27^+^IgG^+^ memory B cell counts (median (IQR) [SD]) |  | 1.06 [-0.50, 1.23] | -1.67 [-1.67, -1.67] | NA [NA, NA] | -0.45 [-0.72, 0.03] | -1.70 [-2.58, -0.57] | -1.31 [-1.83, 0.37] | **-1.36 [-2.03, 0.75]** | **0.050** |
| *RTX therapy regimen* |  |  |  |  |  |  |  |  |  |
| Number of RTX cycles (median [min, max]) ^a^ |  | 1 [1, 6] | 1 [1, 1] | 1[1, 2] | 2 [1, 3] | 1 [1, 4] | 1 [1, 2] | **1 [1, 6]** | **0.004** |
| Number of RTX doses (median [min, max]) |  | 4 [2, 15] | 4 [4, 4] | 6 [1, 8] | 3 [1, 5] | 3 [1, 12] | 3 [2, 4] | **3 [1, 15]** | **0.010** |
| Relative cumulative dose in mg/m^2^ (median [*IQR*]) |  | 1390 [1116, 1509] | 1481 [1343, 1507] | 2200 [783, 2260] | 1097 [750, 1727] | 1140 [761, 1432] | 840 [377, 1885] | **1187 [784, 1521]** | 0.055 |
| *Medication prior to RTX ^b^* |  |  |  |  |  |  |  |  |  |
| Corticosteroids (n (%)) ^c^ |  | 30 (71.4) | 2 (50.0) | 10 (76.9) | 5 (100.0) | 58 (90.6) | 6 (100.0) | **111 (82.8)** | **0.032** |
| Antineoplastic agents (n (%)) ^d^ |  | 3 (7.1) | 0 (0.0) | 12 (92.3) | 0 (0.0) | 54 (84.4) | 0 (0.0) | **69 (51.5)** | **<0.001** |
| Immunosuppressants or immunomodulators (n (%)) ^e^ |  | 30 (71.4) | 2 (50.0) | 10 (76.9) | 5 (100.0) | 59 (92.2) | 6 (100.0) | **112 (83.6)** | **0.017** |
| (Other) biological DMARDs (n (%)) ^f^ |  | 3 (7.1) | 0 (0.0) | 0 (0.0) | 0 (0.0) | 28 (43.8) | 4 (66.7) | **35 (26.1)** | **<0.001** |
| IGRT within five months prior to RTX (n, %) |  | 10 (23.8) | 2 (50.0) | 1 (7.7) | 0 (0.0) | 35 (54.7) | 2 (33.3) | **50 (37.3)** | **0.001** |
| *Medication during or after RTX^b^* |  |  |  |  |  |  |  |  |  |
| Corticosteroids (n (%)) |  | 38 (90.5) | 4 (100.0) | 13 (100.0) | 3 (60.0) | 63 (98.4) | 5 (83.3) | **126 (94.0)** | **0.008** |
| Antineoplastic agents (n (%)) |  | 5 (11.9) | 1 (25.0) | 13 (100.0) | 0 (0.0) | 26 (40.6) | 0 (0.0) | **45 (33.6)** | **<0.001** |
| Immunosuppressants or immunomodulators (n (%)) |  | 38 (90.5) | 4 (100.0) | 13 (100.0) | 3 (60.0) | 63 (98.4) | 6 (100.0) | **127 (94.8)** | 0.005 |
| (Other) biological DMARDs (n (%)) |  | 4 (9.5) | 2 (50.0) | 0 (0.0) | 0 (0.0) | 13 (20.3) | 0 (0.0) | **19 (14.2)** | **0.053** |
| IGRT during or after RTX(n (%)) |  | 18 (42.9) | 4 (100.0) | 6 (46.2) | 0 (0.0) | 56 (87.5) | 1 (16.7) | **85 (63.4)** | **<0.001** |
| Pre-RTX hypogammaglobulinemia (n/total available (%)) |  | 5/32 (15.6) | 0/2 (0.0) | 6/11 (54.5) | 3/4 (75.0) | 14/61 (23.0) | 1/5 (20.0) | **29/115 (25.2)** | **0.026** |

**Supplementary Tables**

Supplementary Table 1. Baseline characteristics by RTX indication

*^a^A cycle was defined as: one or more rituximab doses with no more than 45 days between courses.*

*^b^Medication was classified according to the ATC/DDD Index 2021*

^c^ *Prednisone, dexamethasone, triamcinolone, hydrocortisone*^d^*Cyclophosphamide and other alkylating agents, methotrexate and other antimetabolites, protein kinase inhibitors, miscellaneous*

^e^*Mycophenolic acid, sirolimus, leflunomide, everolimus, cyclosporine, tacrolimus, azathioprine, methotrexate*

^f^*Thymoglobulin, abatacept, infliximab, adalimumab, tocilizumab, miscellaneous*

**P-values were calculated using Chi-squared test for categorical and One-way ANOVA for continuous variables; Kruskal-Wallis and Fisher test were used for non-normally distributed and low (expected) count variables, respectively.*

| RTX indication | Diagnosis | Number of patients |
| --- | --- | --- |
| Autoimmune diseases | Autoimmune cytopenia | 12 |
|  | Autoimmune CNS disease | 8 |
|  | Juvenile Dermatomyositis (JDM) | 5 |
|  | Hematological diseases,  development of inhibitory antibodies^a^ | 5 |
|  | Vasculitis | 3 |
|  | Systemic Lupus Erythematosus (SLE) | 3 |
|  | Autoimmune hepatitis | 2 |
|  | Juvenile Idiopathic Arthritis | 1 |
|  | Miscellaneous | 3 |
| Immunodeficiencies | Immunodeficiencies with autoimmunity | 4 |
| Hematological malignancies | Burkitt lymphoma | 5 |
|  | Non-Hodgkin lymphoma | 3 |
|  | Hodgkin lymphoma | 2 |
|  | Miscellaneous | 3 |
| Nephrotic syndrome | Nephrotic syndrome | 5 |
| Post-HSCT complication | Autoimmune cytopenia | 23 |
|  | EBV infection or reactivation | 21 |
|  | Post-transplant lymphoproliferative disease (PTLD) | 5 |
|  | Humoral transplant rejection | 1 |
|  | Prophylaxis for autoimmune cytopenia^b^ | 9 |
|  | HSCT conditioning regimen^c^ | 5 |
| Post-solid organ transplantation complication | Post-transplant lymphoproliferative disease (PTLD) | 4 |
|  | Humoral transplant rejection | 2 |
| Total |  | 134 |

Supplementary Table 2. RTX indications in detail

^a^*Treatment and/or transfusion related inhibitory antibodies (e.g. factor VIII antibodies)*

*^b^RTX was prophylactically administered pre- and/or post-HSCT. Both were classified as post-HSCT complication.*

*^c^RTX administered pre-HSCT as a part of HSCT conditioning, was also classified as post-HSCT complication.*

|  | **Pre-RTX HG^c^**  *Low IgG*  (n/total available (%)) | **Post-RTX HG**^a^  *Low IgG or receiving IGRT*  (n/total available (%)) | **Persistent HG**^b^ *Low IgG or receiving IGRT (>6 months after RTX)*  (n/total available (%)) | P** |
| --- | --- | --- | --- | --- |
| Autoimmune diseases | 5/32 (14.2) | 10/42 (23.8) | 8/29 (27.5) | 0.450 |
| Immunodeficiencies | 0/2 (0.0) | 4/4 (100.0) | 3/3 (100.0) | 0.480 |
| Hematological malignancies | 6/11 (54.5) | 8/13 (61.5) | 7/10 (70.0) | 0.617 |
| Nephrotic syndrome | 3/4 (75.0) | 5/5 (100.0) | 2/2 (100.0) | 1.000 |
| Post-HSCT complication | 14/61 (23.0) | 43/64 (67.2) | 22/42 (52.4) | **<0.001** |
| Post-solid organ transplantation complication | 1/5 (20.0) | 4/6 (66.7) | 3/5 (60.0) | 0.480 |
| Overall | **29/115 (25.2)** | **74/134 (57.4)** | **46/91 (50.5)** | **<0.001** |
| P* | **0.026** | **<0.001** | **0.026** |  |

**Differences in occurrence of hypogammaglobulinemia between RTX indications, calculated using the Fisher exact test*

***Differences in hypogammaglobulinemia rates pre- and post- RTX, calculated using the McNemar test.*

Supplementary Table 3. Hypogammaglobulinemia (HG) pre- and post-RTX by RTX indication
